# An Analytical System for Single-Cell Metabolomics of Typical Mammalian Cells Based on Highly Sensitive Nano-Liquid Chromatography Tandem Mass Spectrometry

**DOI:** 10.5702/massspectrometry.A0080

**Published:** 2020-03-17

**Authors:** Kohta Nakatani, Yoshihiro Izumi, Kosuke Hata, Takeshi Bamba

**Affiliations:** 1 Division of Metabolomics, Medical Institute of Bioregulation, Kyushu University, 3–1–1 Maidashi, Higashi-ku, Fukuoka 812–8582, Japan

**Keywords:** single-cell analysis, metabolome, hydrophilic metabolites, nano-LC-MS/MS, multiple reaction monitoring

## Abstract

The rapid development of next-generation sequencing techniques has enabled single-cell genomic and transcriptomic analyses, which have revealed the importance of heterogeneity in biological systems. However, analytical methods to accurately identify and quantify comprehensive metabolites from single mammalian cells with a typical diameter of 10–20 μm are still in the process of development. The aim of this study was to develop a single-cell metabolomic analytical system based on highly sensitive nano-liquid chromatography tandem mass spectrometry (nano-LC-MS/MS) with multiple reaction monitoring. A packed nano-LC column (3-μm particle-size pentafluorophenylpropyl Discovery HSF5 of dimensions 100 μm i.d.×180 mm) was prepared using a slurry technique. The optimized nano-LC-MS/MS method showed 3–132-fold (average value, 26-fold) greater sensitivity than semimicro-LC-MS/MS, and the detection limits for several hydrophilic metabolites, including amino acids and nucleic acid related metabolites were in the sub-fmol range. By combining live single-cell sampling and nano-LC-MS/MS, we successfully detected 18 relatively abundant hydrophilic metabolites (16 amino acids and 2 nucleic acid related metabolites) from single HeLa cells (*n*=22). Based on single-cell metabolic profiles, the 22 HeLa cells were classified into three distinct subclasses, suggesting differences in metabolic function in cultured HeLa cell populations. Our single-cell metabolomic analytical system represents a potentially useful tool for in-depth studies focused on cell metabolism and heterogeneity.

## INTRODUCTION

In recent years, the rapid development of next-generation sequencing (NGS) techniques has enabled researchers to acquire single-cell genomic and transcriptomic information. As a result, the importance of heterogeneity in the cell cycle, cell aging, and stochastic biological processes, has become readily apparent.^1)^ For example, NGS-based single-cell transcriptomic analyses revealed that a specific type of lung cells was affected by aging, which subsequently suggested that cholesterol biosynthesis and transcriptional noise due to epigenetic dysregulation were increased in these aging cell population.^2)^ In acute myeloid leukemia, it has been suggested that the heterogeneity of cell types in bone marrow fluid is correlated with malignancy.^3)^ Therefore, understanding the heterogeneity inherent in such biological systems at a single-cell resolution is expected to provide important insights into therapeutic strategies for anti-aging and cancer treatments.

Low-molecular weight metabolites that are essential for biological activities are biosynthesized through metabolic reactions mediated by enzymes. Mass spectrometry is the preferred detection method for metabolome analysis due to its selectivity and sensitivity. In recent years, various technologies have been developed for obtaining data on hydrophilic metabolites at the tissue- or cell-specific level.^4,5)^ Matrix-assisted laser desorption/ionization mass spectrometry (MALDI/MS) under a microscope, imaging mass spectrometry, and live single-cell mass spectrometry (LSCMS) are potential tools for single-cell metabolomics.^6–8)^ However, polar primary metabolites have not yet been identified from single-cells using these techniques. Capillary electrophoresis mass spectrometry (CE-MS) has been developed to identify and quantify hydrophilic metabolites. The advantages of the CE-MS method over MALDI/MS and LSCMS are: i) isomer discrimination by CE separation, ii) decreased effect of the biological matrix by CE separation, and iii) consequently, an increase in the number of metabolites detected. Onjiko *et al.* measured metabolic dynamics during cell division in the early embryo stage of *Xenopus laevis* with a diameter of approximately 1000 μm (523 nL) at the single-cell resolution.^9,10)^ However, successful single-cell metabolomics studies are limited to only relatively large cells. Therefore, developing analytical methods that can be employed to obtain hydrophilic metabolite information from typical mammalian cells (*e.g.*, HeLa cells) with a diameter of approximately 20 μm (4 pL) would be highly desirable.^4)^

Liquid chromatography mass spectrometry (LC-MS) is commonly used for metabolome analysis.^11–13)^ Recently, LC column particles for hydrophilic metabolite analysis using LC-MS have also been developed. For example, a LC-MS method using a pentafluorophenylpropyl (PFPP) column has been used for the practical analysis of hydrophilic metabolites.^14)^ Theoretically, the sensitivity of LC-MS can be increased by lowering the flow rate of the mobile phase, which can be achieved using narrow-diameter LC columns.^15)^ A general nano-liquid chromatography mass spectrometry (nano-LC-MS) system consisting a column with an inner diameter (i.d.) of <100 μm can supply a mobile phase to a nanosprayer at a flow rate of <600 nL/min.^16,17)^ When the ﬂow rate is decreased, smaller droplets will be emitted in the electrospray ionization (ESI) source, which in turn will facilitate the formation of ions in the gas phase. Since the ESI needle can be closer to the MS orifice, ion introduction efficiency will be improved, thus enhancing sensitivity.^17)^ Thus, nano-LC-MS can be used as a potential alternative to CE-MS for single-cell metabolomics. Very few metabolomics applications, but not single-cell metabolomics, have been reported using nano-LC-MS.^18–21)^ The advantages of nano-LC-MS method over CE-MS are: i) large sample injection by trapping metabolites at the top of a nano-LC column (100 nL for nano-LC-MS *vs.* 10 nL for CE-MS^9,10)^), ii) simultaneous analysis of a wide range of hydrophilic metabolites (*i.e.*, cationic and anionic metabolites), and iii) consequently, improved analytical sensitivity and metabolite coverage.

The objective of the present study was to develop an analytical system for single-cell metabolomics of hydrophilic metabolites in typical mammalian cells (HeLa cells) using a combination of living single-cell sampling and PFPP-based nano-liquid chromatography triple-quadrupole mass spectrometry (PFPP-nano-LC-MS/MS) with multiple reaction monitoring (MRM). Using our novel analytical system, we successfully detected 18 relatively abundant hydrophilic metabolites (amino acids and nucleic acid related metabolites) from single HeLa cells (*n*=22). Metabolic heterogeneity at the single-cell level in a dish-cultured HeLa cell population was evaluated.

## EXPERIMENTAL

### Chemicals and reagents

LC-MS-grade water, acetonitrile, and methanol were purchased from Kanto Chemical Co., Inc. (Tokyo, Japan). HPLC-grade chloroform was purchased from Nacalai Tesque, Inc. (Kyoto, Japan). LC-MS-grade formic acid was purchased from Fujifilm Wako Pure Chemical Industries, Ltd. (Osaka, Japan). Authentic standards were obtained from Nacalai Tesque, Inc., Fujifilm Wako Pure Chemical Industries, Ltd., Merck (Darmstadt, Germany), and Honeywell International, Inc. (Morristown, NJ, USA).

### Cell culture and sample preparation

HeLa cells (American Type Culture Collection) were cultured under a humidified atmosphere of 5% CO_2_ at 37°C in Dulbecco’s modified Eagle’s medium (DMEM, Thermo Fisher Scientific, Inc., Waltham, MA, USA) supplemented with 10% (v/v) FBS (Thermo Fisher Scientific, Inc.) and 1% (v/v) Penicillin–Streptomycin Solution (Thermo Fisher Scientific, Inc.) as antibiotics. Cultivated HeLa cells in 6-well plates were harvested at 80% confluency by treatment with Trypsin–EDTA solution for 3 min at 37°C. The Trypsin–EDTA-treated HeLa cells were collected in a 15-mL tube before removing the medium by centrifugation with a swing type rotor at 250 g for 1 min at 20°C. The resulting cell pellets were washed four times with 10 mL PBS before resuspending the washed cell pellets in PBS. After the floating cell suspension in 1 mL PBS was counted using a cell counter (Moxi Z, ASONE Co., Osaka, Japan), 1×10^5^ cells were transferred to a 1.5-mL polypropylene Eppendorf tube.

### Semimicro-LC-MS/MS and nano-LC-MS/MS conditions

A semimicro-LC-MS/MS system used in this study was composed of a Prominence-i LC-2030 HPLC system (Shimadzu Co., Kyoto, Japan) coupled to an LCMS-8060, a triple-quadrupole mass spectrometer (Shimadzu Co.) with a heated ESI source. The LC system was equipped with a binary pump, a temperature-controlled column component, and an autosampler. A Discovery HSF5 column (Merck) with dimensions of 2.1 i.d.×150 mm and a particle size of 3-μm was used for the semimicro-LC separation. A set of mobile phases was used: 0.1% (v/v) formic acid (A) and 0.1% (v/v) formic acid in acetonitrile (B). The gradient conditions were as follows: *t*=0–5 min, 0% B; *t*=5–15 min, 0–40% B; *t*=15–16 min, 40–100% B; *t*=16–20 min, 100% B; *t*=20–20.1 min, 100–0% B; *t*=20.1–25 min, 0% B. The flow rate was set at 0.25 mL/min and the column oven temperature was 25°C. The injection volume was 1 μL. The ESI-MS conditions were as follows: nebulizer gas flow, 2 L/min; heating gas flow, 10 L/min; drying gas flow, 10 L/min; heat block temperature, 400°C; DL temperature, 250°C; and spray voltage, 4.0 kV for positive mode. The MRM parameters for each of 35 hydrophilic metabolites were optimized by flow injection analysis.

Nano-LC-MS/MS analyses were conducted using a Thermo Scientific UltiMate 3000 RSLCnano system (Thermo Fisher Scientific, Inc.) coupled to a LCMS-8060 (Shimadzu Co.) equipped with a nano-LC interface (AMR Inc., Tokyo, Japan) and an HTC-PAL autosampler (CTC Analytics, Zwingen, Switzerland). Packed nano-LC columns integrated with nanosprayers were prepared according to a previously published procedure^18)^ as described below. Tapered nanosprayer tips (a tip outlet diameter of 8−9 μm) were fabricated using a fused-silica capillary (100 μm i.d., 360 μm o.d.) from Polymicro Technologies Inc. (Phoenix, AZ, USA) and a CO_2_ laser-based capillary puller (P-2000, Sutter Instrument Co., Novato, CA, USA). The P-2000 conditions for steps 1–4 steps were HEAT, 225; VEL, 15; and DEL, 138, and step 5 was HEAT, 180; VEL, 15; and DEL, 138. Nanobaume SP-400 column packer system (Western Fluids Engineering, Wildomar, CA, USA) connected to a double-plunger micro pump (KP-22-01A, FLOM Inc., Tokyo, Japan) was employed as the nano-LC columns. A glass reservoir ﬁlled with the slurry of 3-μm Discovery HSF5 (PFPP) particles (Merck) in methanol (5 mg/mL) was placed in a liquid-pressurized column loader cell. The tapered nanosprayer tip was connected to the column loader cell, and the pressure in the cell was increased to prepare a packed nano-LC column of 3-μm particle-size PFPP with dimensions 100 μm i.d.×180 mm. A set of mobile phases was used: 0.1% (v/v) formic acid (A) and 0.1% (v/v) formic acid in acetonitrile (B). The gradient conditions were as follows: *t*=0–9 min, 1% B; *t*=9–19 min, 1–40% B; *t*=19–20 min, 40–99% B; *t*=20–30 min, 99% B; *t*=30–31 min, 99–1% B; *t*=31–45 min, 1% B. The flow rate was set at 600 nL/min and the column temperature was 25°C. The injection volume was 0.1 μL. The nano-ESI-MS conditions were as follows: drying gas flow, 0 L/min; heat block temperature, 400°C; DL temperature, 250°C; and spray voltage, 2.5 kV for positive mode. The nano-LC-MS/MS (MRM) parameters were same as those used for semimicro-LC-MS/MS.

### Single-cell sampling and injection into PFPP-nano-LC-MS/MS system

**Figure 1:**
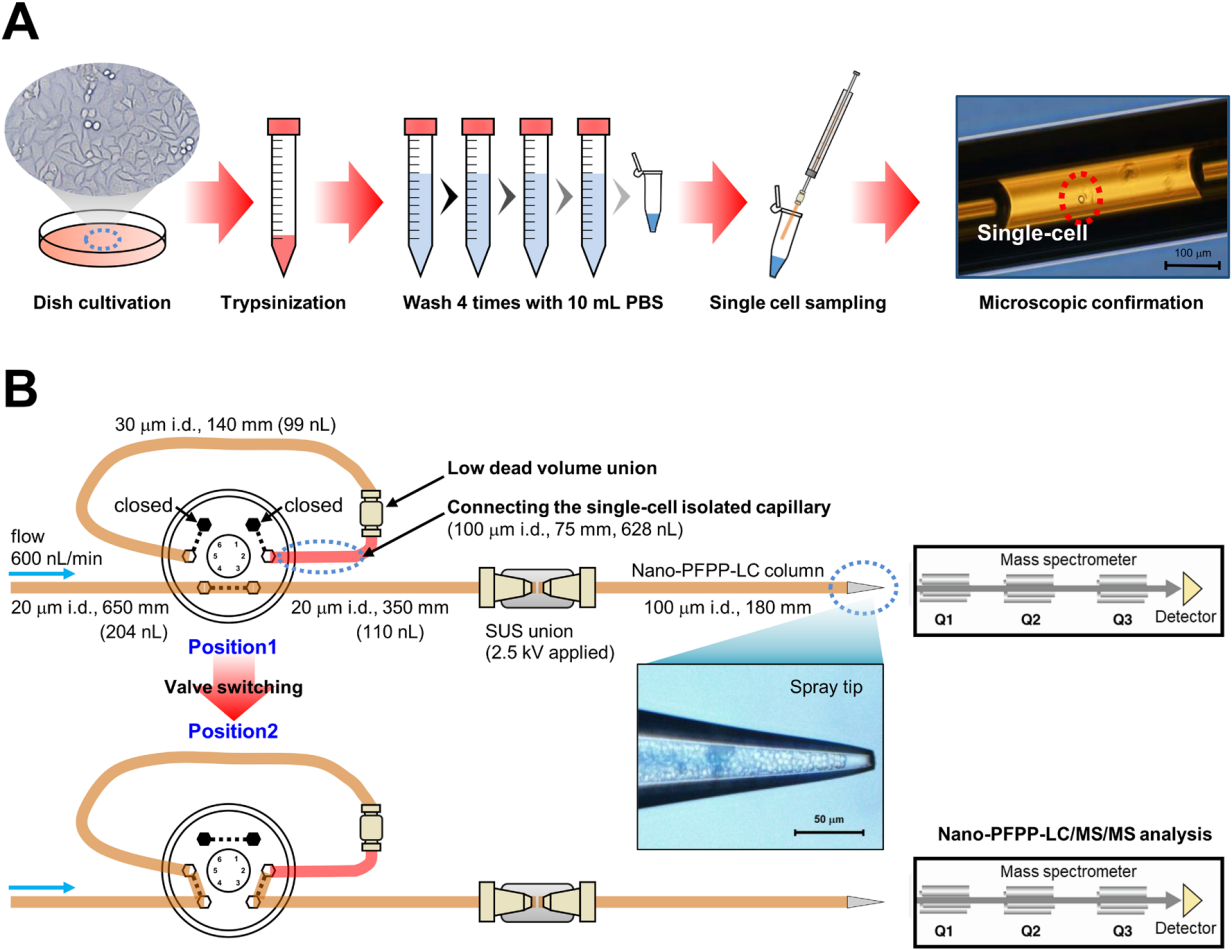
Fig. 1. Overview of single HeLa cell sampling (A) and nano-LC-MS/MS analysis (B).

Living single-cell sampling was performed as described below (Fig. 1A). Before collecting single-cell samples, a fused-silica capillary (100 μm i.d., 360 μm o.d., 75 mm length) was connected to a gas-tight syringe-based nanopipette device^22,23)^ and filled with water as a carrier. By using the cell sampling system, the HeLa cell suspension was aspirated sequentially into the cell sampling capillary as follows: air gap, 50 nL; HeLa cell suspension, approximately 100 nL (10 cells/μL) for 1 cell; and air gap, 50 nL. A PBS solution or the supernatant of the cell suspension were used as blank samples. The single HeLa cell collected in the cell sampling capillary was confirmed by counting the cell using a microscope (IX73, Olympus, Tokyo, Japan) with a color CCD camera (DP73, Olympus). The cell sampling capillary was immediately connected to a sample loop line with a low dead volume union (Fig. 1B). The single-cell sample was then injected to the PFPP-nano-LC-MS/MS system by valve switching.

### Data analysis

Data analyses were performed by LabSolutions version 5.91 (Shimadzu Co.). Box plots were produced by Microsoft Excel 2016. Hierarchical clustering analysis (HCA) using the auto scaling peak area values was performed by the Ward method with a web-based statistical tool, MetaboAnalyst 4.0.^24)^

## RESULTS AND DISCUSSION

### Optimization of MRM conditions for targeted hydrophilic metabolites

In this study, we targeted 35 representative hydrophilic metabolites including amino acids, nucleic bases, nucleosides, and nucleotides that are relatively abundant *in vivo*. To determine the chemical properties of these 35 hydrophilic metabolites, log *P*_ow_, the strongest acidic p*K*_a_, the second strongest p*K*_a_, the strongest basic p*K*_b_, and the second strongest p*K*_b_ were predicted by means of a ChemAxon, MarvinSketch. Based on their molecular/ion distribution at pH 7.0, the 35 targeted hydrophilic metabolites were classified into 4 groups: cationic, anionic, zwitterionic, and uncharged metabolites (Table S1). In addition, the polarity of log *P*_ow_ for the 35 targeted hydrophilic metabolites ranged from −4.88 to −0.57. LC-MS/MS in MRM offers significant advantages regarding selectivity and sensitivity for the analysis of targeted metabolites.^19)^ Parameters for MRM transitions (precursor ion, collision energy, product ion, and pre-quadrupole focusing voltages) were optimized for the 35 hydrophilic metabolites (Table S1).

### Development of a highly sensitive PFPP-nano-LC-MS/MS method

PFPP columns are commonly used as reversed-phase columns that show good performance for separating hydrophilic metabolites.^25)^ We first fabricated nano-LC columns integrated with nanosprayers by packing the PFPP particles (3-μm particle size) into 0.1-mm i.d. fused-silica capillary tubes with a tapered nanosprayer tip (Fig. 1B). To compare the semimicro- (3-μm particle-size PFPP, 2.1 mm i.d.×150 mm) and nano- (3-μm particle-size PFPP, 0.1 mm i.d.×180 mm) LC-MS/MS (MRM) methods, the two columns were used with the same packing material and with a similar length, and the LC and MS/MS parameters were maintained to be the same as far as possible. Diluted standard solutions were used to estimate the limit of detection (LOD), the linear dynamic range, its linearity (*R*^2^ values), and repeatability. The diluted standard solutions were injected into the semimicro-LC-MS/MS (injection volume, 1 μL) or nano-LC-MS/MS (injection volume, 0.1 μL) using an autosampler. Table 1 provides information on the validation of the PFPP-nano-LC-MS/MS method for the 35 hydrophilic metabolites. These results confirm that our developed PFPP-nano-LC-MS/MS method gave peak areas with good repeatability (relative standard deviations, RSDs, <16%; and averaged RSDs, 7.2%) and showed a linear correlation coefficient (*R*^2^) between 0.9800 and 1.0000 for all of the targeted metabolites. In the PFPP-nano-LC-MS/MS system, the LOD for 10 hydrophilic metabolites (phenylalanine, valine, UMP, proline, histidine, tryptophan, GMP, CMP, leucine, and glutamic acid) were in the sub-fmol range, *i.e.*, in the range of 20–950 amol. Using the PFPP-nano-LC column, the baseline separation of isoleucine and leucine structural isomers was also obtained (Table 1).

**Table 1:** Table 1. Performance of the PFPP-nano-LC-MS/MS method.

Standard	RT (min)	RSD of peak area (*n*=3) (%)	Linear range (fmol)	Correlation factor (*R*^2^)	LOD (fmol)^a^	Sensitivity improvement (nano-LC/semimicro-LC)	Amount per injection used for sensitivity comparison (pmol)
UMP	3.0	4.2	1–1000	0.9959	0.34	58	1
GMP	3.1	6.0	1–1000	0.9800	0.51	26	1
dTMP	3.2	9.1	1–1000	0.9930	1.0	45	1
Asparatic acid	3.3	3.8	10–1000	0.9971	7.3	11	1
Glutamine	3.5	2.0	1–1000	0.9979	1.3	35	1
Serine	3.5	4.6	10–1000	0.9969	4.8	7	1
Glutamic acid	3.5	6.5	1–1000	0.9996	0.95	21	1
Asparagine	3.5	3.7	100–1000	1.0000	22	13	1
Cysteine	3.6	3.7	100–1000	1.0000	3.6	15	1
Threonine	3.6	2.3	1–1000	0.9970	1.3	17	1
Alanine	3.6	15.4	100–1000	1.0000	14	7	1
Glycine	3.6	4.9	100–1000	1.0000	38	9	1
Proline	3.7	5.9	1–1000	1.0000	0.43	27	1
CMP	3.7	15.4	0.1–1000	0.9916	0.56	45	1
Histidine	4.6	12.3	1–1000	0.9982	0.45	68	1
Lysine	4.6	15.4	10–1000	0.9992	10	30	1
AMP	4.6	10.7	10–1000	0.9999	1.3	36	1
Arginine	5.1	5.4	10–1000	0.9997	2.5	10	1
Cytosine	5.1	5.6	10–1000	0.9999	4.9	3	1
Uridine	5.6	16.2	10–1000	1.0000	3.7	55	1
Guanine	6.2	15.7	100–1000	1.0000	32	3	1
Reduced glutathione	6.2	7.8	100–1000	1.0000	15	16	1
Valine	6.4	4.8	0.1–100	0.9997	0.043	8	0.1
Methionine	7.1	7.3	10–1000	0.9992	8.7	7	1
Adenine	7.6	6.4	10–1000	0.9997	9.2	3	1
Cytidine	8.2	16.2	0.1–1000	0.9999	1.5	34	1
Guanosine	10.2	7.5	1–1000	0.9999	5.7	9	1
Oxidized glutathione	11.1	7.8	10–1000	0.9989	1.1	132	1
Isoleucine	12.0	4.2	1–1000	1.0000	1.1	14	1
Thymidine	13.4	2.7	10–1000	0.9998	2.3	23	1
Leucine	13.4	4.4	1–1000	0.9999	0.75	7	1
Adenosine	15.1	2.4	0.1–1000	0.9999	1.6	13	0.1
Tyrosine	18.2	7.1	10–1000	1.0000	25	10	1
Phenylalanine	18.3	2.6	1–1000	1.0000	0.020	61	0.1
Tryptophan	22.5	2.1	1–1000	0.9985	0.49	35	0.1
Average	—	7.2	—	0.9983	6.4	26	—

^a^ LOD was estimated based on *S*/*N*=3.

To compare the results for the semimicro-LC-MS/MS and nano-LC-MS/MS methods, the sensitivity of detection is the most important issue. Because the noise intensity of some MRM transitions was zero in the semimicro-LC-MS/MS results, it was difficult to accurately compare the sensitivities of the two methods based on signal-to-noise (*S*/*N*) ratios. Therefore, the sensitivity of the two methods was compared using the peak intensity ratio when the same amount of compound was injected (Table 1). The nano-LC-MS/MS method demonstrated a sensitivity of 3–132-fold (average value, 26-fold) greater than that of the semimicro-LC-MS/MS method. This confirms that downsizing the column i.d. resulted in an improved sensitivity.

### PFPP-nano-LC-MS/MS-based single-cell metabolomic analytical system

To apply the highly sensitive PFPP-nano-LC-MS/MS method to single-cell metabolomics, we designed and developed a living single-cell sampling system with a nanopipette device (Fig. 1A). Before single-cell sampling, a fused-silica capillary (100 μm i.d., 360 μm o.d., 75 mm length) was connected to a nanopipette device. Single HeLa cells could be aspirated into the sampling capillary using the sampling device. The volume of the collected single-cell suspension was approximately 100–200 nL. After confirming under a microscope that a single cell was in the capillary tube, the capillary was immediately connected to a sample loop line with a low dead volume union (Fig. 1B). The single cell was then introduced into the PFPP-nano-LC-MS/MS system by switching the valve from position 1 to position 2 (Fig. 1B). After switching the valve, the introduced HeLa cell was mixed with an acidic mobile phase (pH 2.8) and was then subjected to the pressure of the nano-LC column back pressure (approximately 40 MPa). Eventually, a nano-ESI voltage (2.5 kV) was applied to the cell at the SUS union (Fig. 1B). These steps instantaneously disrupted the cell membrane, allowing intracellular metabolites from a single cell to be injected into the system. The extracted single cell metabolite solution (<100 nL) was trapped at the top of the PFPP-nano-LC column and eluted for separation with a gradient elution and then ionized at the sprayer tip (Fig. 1B). The total processing time from the trypsin treatment to cell washing, single-cell sampling, and sample loading was 25 min. The time from single-cell sampling to sample loading was approximately 5 min.

**Figure 2:**
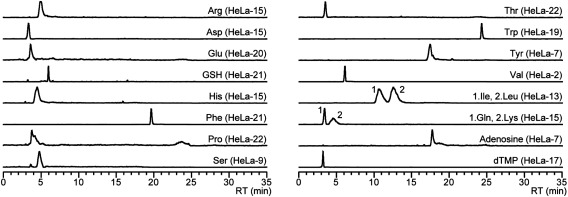
Fig. 2. PFPP-nano-LC-MRM chromatograms of metabolites detected from single HeLa cells. A total of 16 amino acids and 2 nucleic acid-related metabolites were detected.

**Figure 3:**
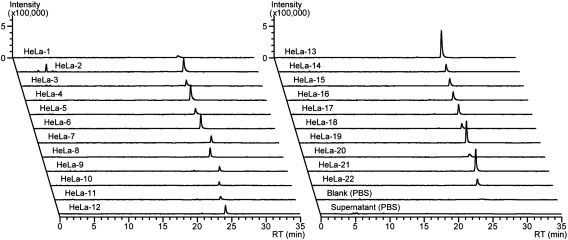
Fig. 3. PFPP-nano-LC-MS/MS chromatograms of tryptophan obtained from single HeLa cells (*n*=22). A PBS solution (blank) and the supernatant of the cell suspension (supernatant) were used for evaluating carryover or cross-contamination.

We successfully detected 18 relatively abundant hydrophilic metabolites (16 amino acids and 2 nucleic acid related metabolites) from a single HeLa cell (HeLa-1–HeLa-22) by the single-cell sampling and PFPP-nano-LC-MS/MS system (Fig. 2). Based on the “level 1-identified metabolites” criteria defined by the Metabolomics Standards Initiative (MSI),^26)^ the peaks of target metabolites from single cells were identified by comparing their chromatographic retention times and mass spectrometric MRM transitions with that of authentic standards. Our PFPP-nano-LC-MS/MS method enabled the separation and detection of a wide range of hydrophilic compounds, including cationic- (*e.g.*, educed glutathione), anionic- (*e.g.*, dTMP), zwitterionic- (*e.g.*, tryptophan), and uncharged (*e.g.*, adenosine) metabolites from single HeLa cells (Tables S1). Figure 3 shows PFPP-nano-LC-MRM chromatograms of tryptophan obtained from single HeLa cells. Tryptophan was detected in every single cell sample, suggesting that intracellular levels of tryptophan vary widely. A single-cell sample (HeLa-22), a PBS solution sample (blank), and the supernatant of the cell suspension sample (supernatant) were analyzed sequentially, confirming that carryover or cross-contamination were not issues in the single-cell analysis obtained in this study (Fig. 3).

### Metabolic profiling of single HeLa cells

**Figure 4:**
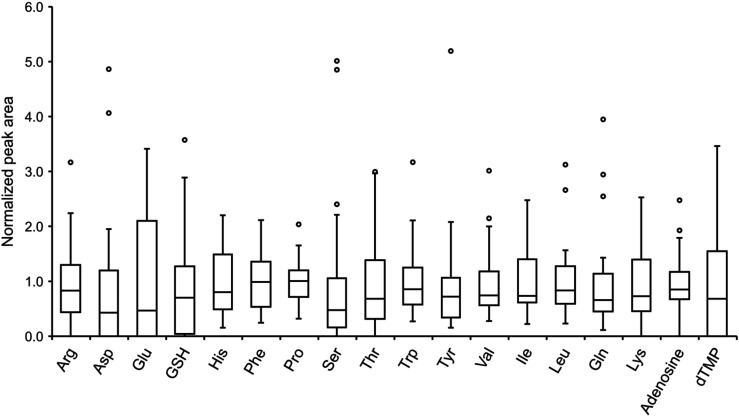
Fig. 4. Variation in 18 hydrophilic metabolites detected from single HeLa cells (*n*=22). The peak area of each metabolite was normalized to the average peak area of each metabolite. The box signifies the first quartile (Q1) and third quartile (Q3), and the median. Interquartile range (IQR) is from Q1 to Q3. The point outside the Q3+1.5×IQR or Q1−1.5×IQR were plotted as outlier points. The whisker signifies the maximum and minimum points.

**Figure 5:**
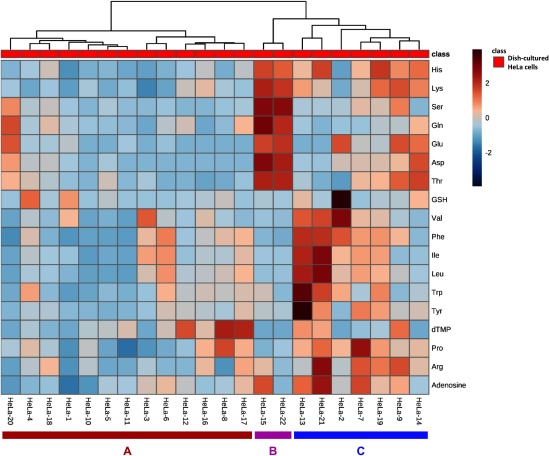
Fig. 5. HCA results for 22 single HeLa cells using peak areas of the detected metabolites. The horizontal axis indicates single-cell samples (HeLa-1–HeLa-22) and the vertical axis indicates metabolites. The HCA conditions were as follows: distance, Euclidean; clustering algorithm, Ward; and metabolome data, mean-centered and divided by the standard deviation of each variable.

The peak area for each of the 18 metabolites from single HeLa-cells were normalized by the averaged peak area of each metabolite, and the variation of each metabolite in single-cells is shown in Fig. 4. In this study, the metabolite with the largest variation in single-cells was tyrosine, the maximum of which was 5.2 times the mean and the minimum of which was 0.15 times the mean. The metabolite with the smallest fluctuation in single cells was proline, the maximum of which was 2.0 times the mean and the minimum of which was 0.32 times the mean. These results suggest that single cells in a population of cultured HeLa cells contained heterogeneous levels of metabolites. After autoscaling the metabolite data from 22 single HeLa cells, we performed HCA using the Ward method (Fig. 5). Single HeLa cells could be mainly classified into three subclasses (A, B, and C) based on their metabolic similarities. Several subclasses of cultured HeLa cell populations may reflect cell cycle differences and metabolic diversity.

In summary, we report on the development of a system for analyzing single-cell metabolomics of hydrophilic metabolites in typical mammalian cells using a combination of living single-cell sampling and PFPP-nano-LC-MS/MS. Using this system, a total of 18 hydrophilic metabolites were successfully identified by analyzing a total of 22 single HeLa cells. In addition, HCA results suggest that there were subclasses showing metabolic similarities among these 22 single HeLa cells. To our knowledge, this represents the first report to describe metabolic heterogeneity at the single-cell level in a dish-cultured HeLa cell population. The analytical system developed in this study is a new tool for single-cell metabolomic analysis and thus will facilitate future research in tumor heterogeneity.

## Notes

Mass Spectrom (Tokyo) 2020; 9(1): A0080
